# Automatic photoacoustic monitoring of perinatal brain hypoxia with superior sagittal sinus detection

**DOI:** 10.1117/1.JBO.30.7.076004

**Published:** 2025-07-11

**Authors:** Baichuan Jiang, Ernest Graham, Mathias Unberath, Russell H. Taylor, Raymond C. Koehler, Jeeun Kang, Emad M. Boctor

**Affiliations:** a Johns Hopkins University, Department of Computer Science, Baltimore, Maryland, United States; b Johns Hopkins Medical Institute, Department of Gynecology and Obstetrics, Baltimore, Maryland, United States; c Johns Hopkins Medical Institute, Department of Anesthesiology and Critical Care Medicine, Baltimore, Maryland, United States; d Johns Hopkins University, Department of Electrical and Computer Engineering, Baltimore, Maryland, United States

**Keywords:** perinatal health, brain monitoring, photoacoustic imaging, oxygen saturation measurement, deep learning

## Abstract

**Significance:**

Despite advances in perinatal medicine over decades, perinatal hypoxic-ischemic encephalopathy (HIE) remains a significant cause of fetal cerebral palsy and can lead to other severe medical sequelae or death. Therefore, it is highly desirable to effectively detect brain hypoxia during labor and postnatally for HIE management.

**Aim:**

We recently validated the feasibility of transcranial photoacoustic (PA) imaging for oxyhemoglobin saturation measurement at the superior sagittal sinus ($O_{2}{\mathrm{Sat}}_{\mathrm{ss}}$) in the neonatal piglet brain, at which overall oxygen supply status can be reflected as a primary collective vein. We aim to automate the PA-based workflow of at-risk subject detection and enable fully autonomous and continuous perinatal monitoring.

**Approach:**

We proposed a two-step algorithm that focuses on the most informative region of the brain for oxygenation status, the superior sagittal sinus (SSS). First, a convolutional neural network (U-Net) is trained to detect the location of SSS in the coronal cross-section PA images. Then, an optimized region of interest patch around the predicted SSS location is cropped from the spectral unmixed image and averaged as the $O_{2}{\mathrm{Sat}}_{\mathrm{ss}}$ measurement. A confidence score can be computed for the measurement via Monte Carlo dropout (MCD), which infers the prediction uncertainty for better clinical decision-making.

**Results:**

The algorithm was evaluated on an *in vivo* piglet brain imaging dataset containing 84 independent experimental settings from 10 piglet subjects. A 10-fold leave-one-subject-out cross-validation experiment reports 85.2% sensitivity and 93.3% specificity for healthy/hypoxia classification with an R-squared value of 0.708 and a confidence score of 94.06% based on MCD computation, well agreed with our ground-truth given by blood gas measurements.

**Conclusions:**

The proposed automatic $O_{2}{\mathrm{Sat}}_{\mathrm{ss}}$ monitoring solution demonstrated a hypoxia detection capability comparable to the human expert manual annotation on the same task. We concluded with high feasibility for a noninvasive PA-based continuous monitoring of the perinatal brain.

## 1 Introduction

Perinatal hypoxic-ischemic encephalopathy (HIE) is estimated to affect approximately one to three per 1000 live full-term births and can lead to severe neuropsychological sequelae or death. It was reported that ∼25% of the affected newborns will develop severe sequelae, including cerebral palsy, mental retardation, epilepsy, or increased hyperactivity, and 15% to 20% will die in the postnatal period.^1^

Several modalities have been proposed to diagnose and monitor perinatal HIE. During the intrapartum period, an electronic fetal heart rate monitor has been a standard of care to monitor fetal health. Despite its substantial contribution to the reduction of fetal mortality, it has brought soaring healthcare costs with limited implications to monitor fetal brain health: a fivefold increase in the Cesarean section rate over a period of 30 years since the 1970s, the false-positive rate for childhood neurologic injury prediction approaches 99.8%, whereas the rate of cerebral palsy has not decreased.^2^^–^^4^

During the postpartum period, magnetic resonance imaging (MRI) has superior anatomic resolution and sensitivity for acute ischemia, but it has limited sensitivity for detecting injury within the first 6 h after the insult when neuroprotective therapy needs to be administered.^5^^,^^6^ Moreover, MRI is generally not performed on newborns until 7 to 10 days after birth due to the difficulty in transporting the newborn with HIE out of the intensive care unit (ICU) and avoiding using metal equipment near magnets.^7^ Ultrasound (US) can be used within the ICU and is good at detecting intracranial bleeding, but it generally cannot distinguish normal from hypoxic brain tissue.^8^ Bio-photonic modalities such as near-infrared spectroscopy (NIRS) and diffuse-optical tomography (DOT) have shown their potential for the noninvasive sensing of cerebral oxygenation level and hemodynamic changes in the neonatal brain.^9^^,^^10^ However, both modalities have low spatial resolution (5 to 10 mm).^11^ It can be challenging to correctly measure arterial blood oxygenation in fetal application with neighboring veins present in the same resolution cell.^12^ Therefore, a new modality for effectively detecting brain hypoxia during labor and postnatally is highly desirable. With such a diagnostic tool, rapid decision-making during labor and better management of neonates can be enabled.

Photoacoustic (PA) imaging is a noninvasive, radiation-free hybrid imaging modality, quantifying acoustic pressure originated from transient thermoelastic tissue expansion upon nonionizing pulsed excitations of multiwavelength light. The laser light can penetrate deep into the tissue, and each tissue composition has a unique absorption spectrum, which enables visualizing molecular light absorbance at the acoustic spatial resolution (approximately hundreds of micrometers) and imaging depth up to several centimeters. Multiwavelength imaging also provides quantitative tissue characterization based on their optical absorption spectra.^13^ There have been many preclinical/clinical advances using PA imaging.^14^^–^^20^ Among them, quantifying blood oxyhemoglobin saturation ($O_{2}{\mathrm{Sat}}_{\mathrm{ss}}$) has been considered the most prominent clinical application as $O_{2}{\mathrm{Sat}}_{\mathrm{ss}}$ being one of the most crucial physiological parameters.^18^^–^^20^

Research efforts have been made to automate the estimation of $O_{2}\mathrm{Sat}$ and achieve real-time hypoxia detection with PA imaging^21^ with the advances in the field of artificial intelligence (AI), especially deep learning.^22^ Research studies^23^^–^^26^ showed that convolutional neural networks (CNNs) can be used to estimate blood oxygenation using the spectra of 2D or 3D PA images. However, these methods are being trained and evaluated exclusively on simulated data, which requires further validation as there is a large domain gap between the experimental PA images and simulated data.^21^ Gröhl et al.^27^ demonstrated that fully connected neural networks can perform learned spectral decoloring (LSD) and yield more accurate oxygenation estimation from single-pixel spectra than the linear unmixing approach on *in vivo* experiment data. In addition, the multiple illumination (MI) technique can be combined with LSD to further improve the $O_{2}\mathrm{Sat}$ estimation.^28^^,^^29^ However, human examination of the spectral unmixed images is still needed to identify the at-risk subjects as the image field of view usually contains arteries, veins, and other biological tissues. Moreover, to differentiate potential fetal/neonatal HIE, it is important to consider both the oxygen saturation and the time duration of low $O_{2}\mathrm{Sat}$ events. In this regard, expecting clinicians’ efforts to frequently examine the spectral unmixed images during labor for 6 to 10 h or during intensive care for days is impractical. Therefore, further development and validation of an automation workflow for continuous fetal/neonatal brain hypoxia monitoring is desired.

In this work, we present a fully automatic PA-based hypoxia detection workflow evaluated on *in vivo* preclinical PA imaging data with blood–gas measurement as ground truth using a neonatal piglet model, which resembles neuroanatomy and neurophysiology of term human babies. The key novelty of the proposed workflow is the seamless integration of image processing algorithms with the knowledge of fetal brain anatomy: instead of training an end-to-end learning algorithm for inferring brain hypoxia from the entire PA image, we focus on the most informative region of the brain oxygenation status, the superior sagittal sinus (SSS), at which the overall oxygen supply can be reflected as a large collective cerebral vein of the brain that drains the bilateral cerebral hemispheres and receives blood from multiple draining vessels.^30^ Specifically, we assume a coronal plane cross-sectional PA image can be acquired continuously to evaluate fetal/neonatal brain health. Recent studies, including our previous work, have validated the basic PA sensing feasibility to estimate the $O_{2}{\mathrm{Sat}}_{\mathrm{ss}}$ level of sheep and piglets transcranially.^19^^,^^31^^–^^33^ Perinatal patients allow the technology to be less invasive and more cost-efficient because of the presence of open fontanelles that allow semi-transparency to acoustic and optical penetration. Once the coronal plane spectral PA image of the brain is obtained, following our envisioned translational implementation plan outlined in Sec. 5, a U-Net-like^34^ convolutional neural network can be trained to localize the region of interest (ROI) patch over SSS within the PA image. The same ROI location on the spectral unmixed $O_{2}\mathrm{Sat}$ map is used to calculate the $O_{2}{\mathrm{Sat}}_{\mathrm{ss}}$ level representing the overall brain oxygenation status. Moreover, a confidence score can be computed for each estimation by running Monte Carlo dropout (MCD) for the proposed algorithm, providing a way to infer the result uncertainty and urgency to plan follow-up courses of clinical actions.

We will first describe the algorithm workflows in Sec. 2, where the setups of a two-step workflow and an end-to-end learning-based workflow for comparison are presented. Then, in Sec. 3, we show the training and evaluation results followed by results discussions in Sec. 4. In Sec. 5, we conclude with the summary and development plans for future clinical translation.

## 2 Methodology

### 2.1 *In Vivo* Piglet Imaging Dataset Preparation

**Fig. 1:**
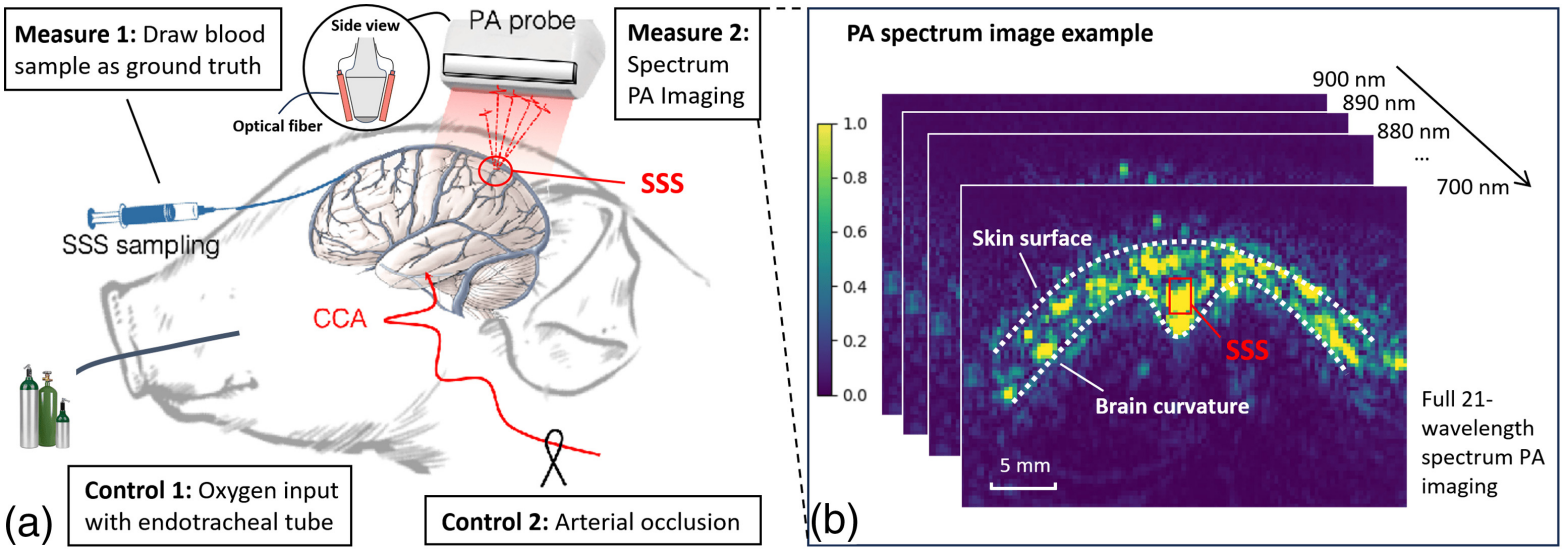
*In vivo* piglet experiment with controlled oxygenation and multiwavelength photoacoustic imaging. (a) Experiment setup with oxygen input controls and multimodal measurements. (b) 21-wavelength photoacoustic imaging example where brain and skin surface structure are clearly visible with appropriate dynamic range settings.

Newborn piglets have skull and brain development status comparable to those of term human newborns, including anatomical and neurological similarities as well as the vulnerability to hypoxia and ischemia.^35^^,^^36^ Therefore, newborn piglets are ideal animal subjects to evaluate the proposed technology to be used for neonatal brain hypoxia monitoring. Figure 1 shows our *in vivo* piglet brain PA imaging setup.^19^ During the experiment, each 3 to 5-day-old piglet was anesthetized and maintained with a controlled level of a fraction of inspired oxygen (${\mathrm{FiO}}_{2}$) ranging from 1.0 to 0.08 by blending the balance of oxygen and nitrogen delivered to an endotracheal tube or introducing arterial occlusion. The SSS was catheterized, and the blood was drawn at each ${\mathrm{FiO}}_{2}$ level for the ground-truth blood–gas measurement of $O_{2}{\mathrm{Sat}}_{\mathrm{ss}}$, which is used to compare against the estimated $O_{2}{\mathrm{Sat}}_{\mathrm{ss}}$ from our proposed automatic workflow.

A bifurcated fiber optic bundle (as shown in Fig. 1), each branch 40 mm long and 0.88 mm wide and attached to the sides of the PA probe, was used for laser pulse delivery.^19^ After each change of ${\mathrm{FiO}}_{2}$, NIR PA signals (700 to 900 nm at a 10-nm increments) were generated at 20 Hz pulse repetition frequency using a tunable optical parametric oscillator (Phocus Inline, Opotek Inc., Carlsbad, California, United States) equipped with the second-harmonic (532 nm) Nd:YAG pulse laser system and recorded with a linear US array (L14-5 from Ultrasonix Medical, 128 elements, 7MHz center frequency with 60% fractional bandwidth, connected to the SonixDAQ system from Ultrasonix Medical Corp., Canada) placed along the coronal plane of the piglet’s head, which included SSS at the lateral center of the FOV. In this setup, the SSS was located at ∼20  mm depth distance from the array to be at the elevation focus. The energy density on the skin surface was $3.5\;{\mathrm{mJ}/\mathrm{cm}}^{2}$ at 720 nm. The energy density can be estimated at each wavelength using the laser spectrum information and two noise models, as presented in an earlier work.^16^ We measured the energy for each wavelength and corrected them before the spectral unmixing. We note that in our experiments, neonatal piglets have already developed cranium with 1 to 4 mm thickness, which will give much more signal attenuation than what we were expecting with fontanelle in fetuses or neonates. We did not try mimicking fontanelle as it will include an invasive procedure and bleeding in the area will contaminate our imaging. Therefore, we expect that the photoacoustic image quality will be higher in transfontanelle photoacoustic imaging, which increases our translational feasibility.

A dataset of 84 data points was collected from a total of 10 piglets. Please note that the total number of data points is not a multiple of 10 because we gradually reduce the input ${\mathrm{FiO}}_{2}$ level to extremely low,^19^ and some piglet subjects died sooner than others, which results in a varied number of data points for each subject. The number of data points corresponding to subjects 1 to 10 is listed as follows: [10, 5, 6, 9, 11, 9, 8, 11, 6, 9]. Each data point was obtained at different ${\mathrm{FiO}}_{2}$ levels and consists of (1) PA spectrum images at the 21 wavelengths, (2) $O_{2}{\mathrm{Sat}}_{\mathrm{ss}}$ value measured from SSS blood gas reading, and (3) SSS location (*x*, *y*) annotated by experts based on the visual identification of the brain/skull M-shape structures of the PA images based on the anatomical knowledge, as shown in Fig. 1(b). More details about the data acquisition protocol can be found in Ref. 19.

**Fig. 2:**
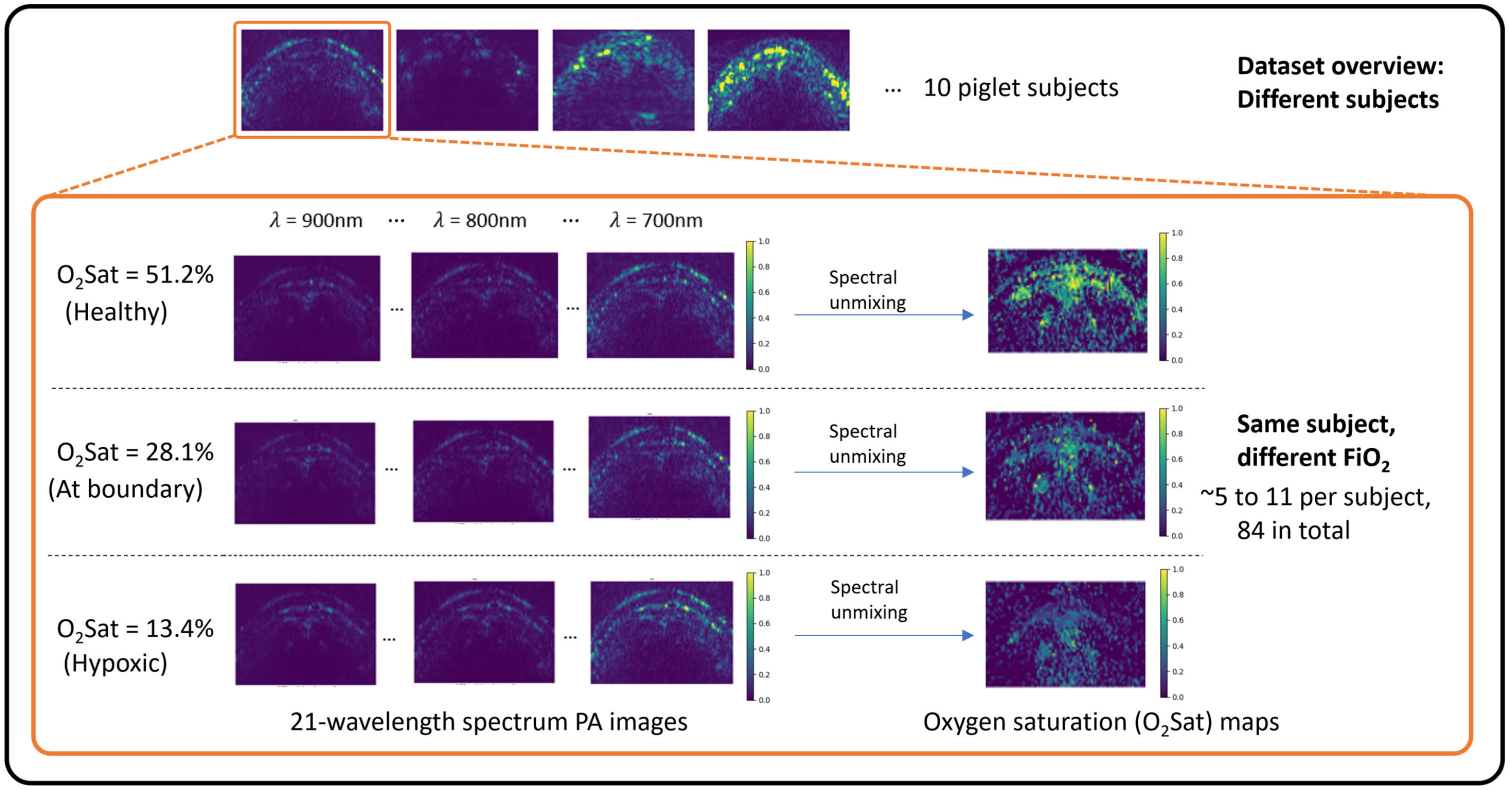
Dataset overview. For different piglet subjects, large intensity and distribution variations can be seen: the top row shows images with a dynamic range min-max normalized. For different ${\mathrm{FiO}}_{2}$ data points of the same piglet subject, the raw photoacoustic spectrum images are visually similar unless spectral unmixing is performed.

An overview of the collected PA spectrum image dataset is shown in Fig. 2. In addition to the relatively small dataset size, there are several challenges inherent in this dataset, which are also expected in practical clinical diagnostics and monitoring. First, overall datasets contain inevitable image appearance variations due to manual imaging probe placement and anatomical differences among the subjects. Second, the range of the absolute PA signal intensities and $O_{2}{\mathrm{Sat}}_{\mathrm{ss}}$ can have large variations for different subjects even on the same ${\mathrm{FiO}}_{2}$ level because of obvious differences in acoustic/optical tissue interactions and biological processes for each subject. Therefore, an effective and consistent way to process divergent image representations in overall datasets is desired, which is presented later in Sec. 2.2.1.

### 2.2 Two-Step SSS Localization-Based Oxygenation Level Estimation Workflow

**Fig. 3:**
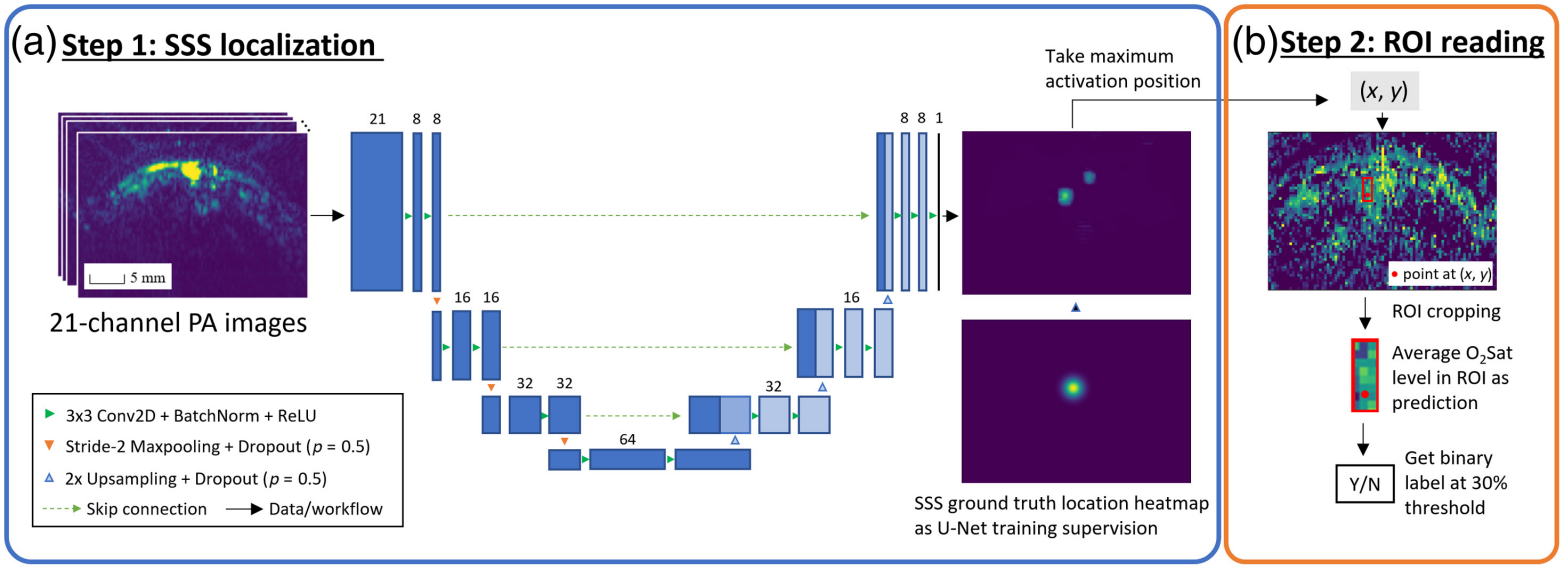
Overview of the proposed algorithm workflow: the anatomical landmark of SSS is first identified, and region of interest (ROI) patch around the SSS in the spectral unmixed $O_{2}\mathrm{Sat}$ map is used as oxygen level prediction.

Given the challenges, it may be unwise to develop an end-to-end learning method for direct $O_{2}{\mathrm{Sat}}_{\mathrm{ss}}$ estimation without the massive amount of data. Instead, we developed a two-step approach that identifies the brain and vasculature structure of each piglet in the dataset as the intermediate step, as shown in Fig. 1(b). The overview of our proposed algorithm is presented in Fig. 3. In the first step, we train a U-Net to localize the SSS position. Then, in the next step, we take the identified SSS location to crop an ROI patch in spectral unmixed $O_{2}\mathrm{Sat}$ map and compute the average $O_{2}\mathrm{Sat}$ value within the ROI over the SSS as the predicted $O_{2}{\mathrm{Sat}}_{\mathrm{ss}}$ level.

#### 2.2.1 Anatomical landmark (SSS) detection with U-Net

The proposed landmark detection U-Net architecture (SSSLocNet) is shown in Fig. 3. It takes the 21-channel raw PA images as input and generates a 1-channel SSS localization heatmap as output. The whole architecture contains four resolution levels, with 64 feature channels at the deepest level. Dropout layers^37^ were introduced to regularize the network training and improve generalizability for unseen piglet subjects. For training the SSSLocNet, the ground-truth SSS heatmap is generated from the expert annotation of a point to represent the vessel center, which is generated by applying a Gaussian filter of kernel size 3 to the binary expert annotation and then performing a min-max normalized between 0 and 1, as shown in Fig. 3. Given the challenges listed in Sec. 2.1, important training techniques described below were applied to avoid overfitting and achieve the best cross-subject generalization.

##### Dynamic range rectification

**Fig. 4:**
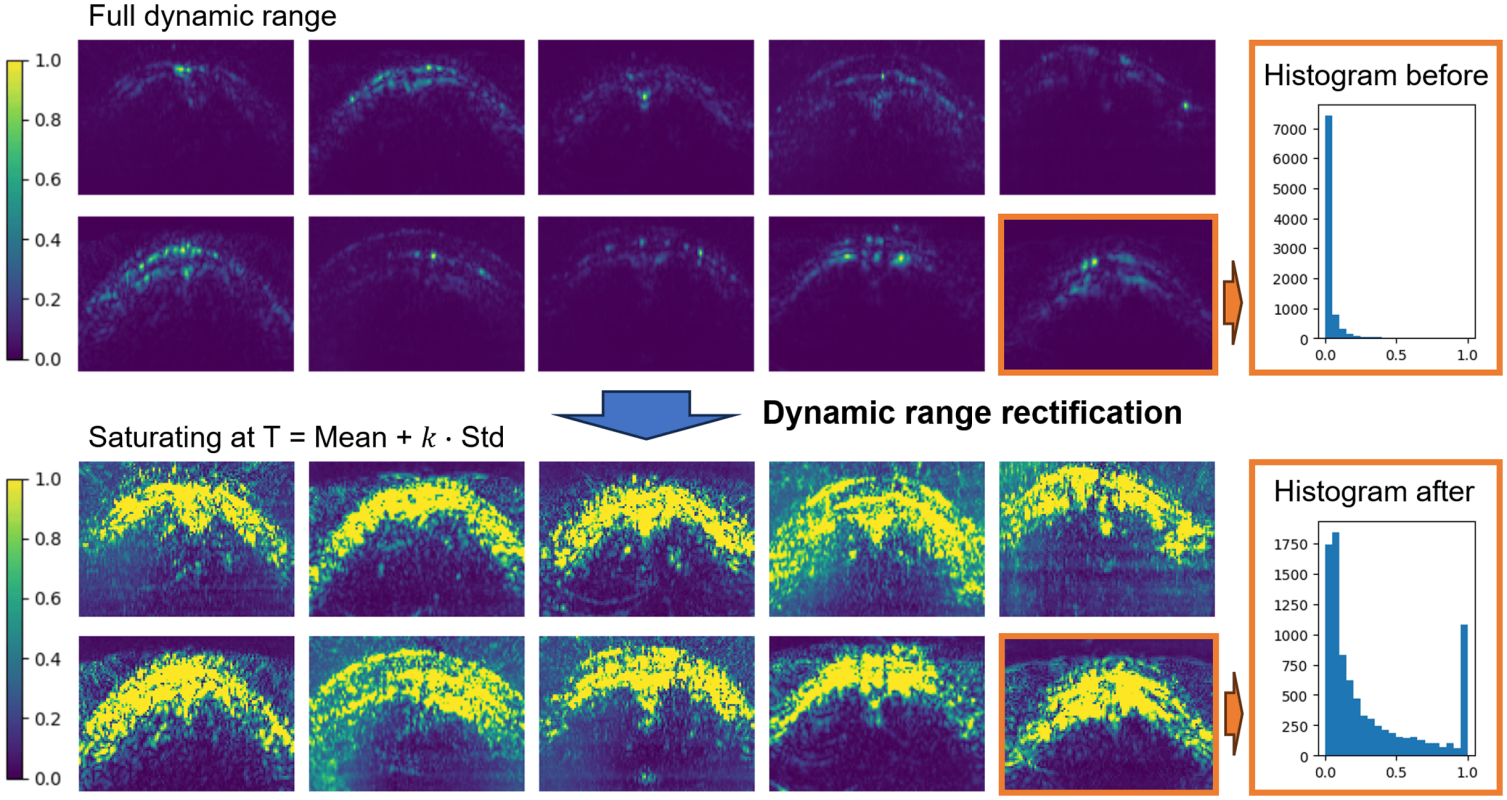
Raw PA image dynamic range rectification to enhance the structural information present in the raw PA signals, such that maximum intensity is saturated at spectral average intensity plus k standard deviation of full spectrum intensities, where k is empirically chosen as 0.5 during test time, and varying between 0 and 1 for training augmentation. Example images from each of the 10 piglet subjects are presented.

The absolute values within the reconstructed PA images can range from 1750 to 2,300,000 in arbitrary units (AU), and anatomical structure information can be dominated by high intensity features. To enhance the structural information present in the raw PA images, e.g., the brain curvature, the dynamic range of the PA images from each data point was rectified such that highest intensity value was saturated at a threshold T, which is set as the average intensity plus k standard deviation of the full spectrum images, as shown in Fig. 4, where k is empirically chosen as 0.5. The intuition behind this procedure is that the brain curvature and skull/skin surface shape in the PA images (as shown in Fig. 1) are in general represented by the cortical vein structures, surrounding the brain surface and converging onto the SSS at the midline, forming the “M-shape” appearance in the image. During dataset annotation, our expert also follows the same mentality to identify the SSS ROI. These signal intensities of the cortical veins are typically irregular depending on the unpredictable structure and imaging configuration. Therefore, by rectifying the dynamic range with intensity saturation, structural information will be enhanced as both the micro and major vessels are equally presented to highlight the brain curvature for easier SSS localization. Although this procedure may result in some loss of information, it helps focus the network training on the specific task of SSS localization without requiring large amounts of data. The threshold value is chosen as the average intensity plus k standard deviation such that it saturates the extreme intensities while preserving the majority of the original intensity information. The intact signal intensity information will be better exploited in step 2 of the algorithm where spectral unmixing is relied on to estimate the true oxygen level.

##### Data augmentation

Extensive data augmentation is needed to avoid overfitting during training, and the following augmentation methods were employed: (1) Dynamic range rectification augmentation: at the training time, the intensity saturation coefficient k was randomly chosen within the range of [0, 1.0]. This process aims to improve the prediction robustness against variations in PA signal intensity distribution. (2) Geometric augmentations were applied, including horizontal flip of the image, grid distortion, random image shifting, rotation, and scaling. This treatment was designed to help generalize the network to unseen brain anatomy and was implemented with the Albumentation package.^38^ (3) Input channel augmentation: with a 50% augmentation probability, instead of using the full 21-wavelength spectrum image as the input during training, we will randomly select five wavelengths of channels out of the 21 spectrum and mask the rest of the channels with zeros. Because the optical absorption ratio of different types of molecules is highly dependent on the wavelength, to avoid selecting the five channels with neighboring wavelengths, we divide the full spectrum into five bins, i.e., [700 to 730 nm], [740 to 770 nm], [780 to 820 nm], [830 to 860 nm], and [870 to 900 nm], and randomly draw one wavelength from each bin to be the nonzero channels. This augmentation is essentially applying channel dropout at the input but with careful treatment to preserve the original spectrum information, as nearby wavelengths may contain similar PA responses. During inference time, all 21 wavelengths of data will be used. The hyperparameters of the network training are set according to Table 1 (SSSLocNet).

**Table 1:** Network training hyperparameter settings.

Parameter name	SSSLocNet	$O_{2}\mathrm{Sat}$ PredNet
Batch size	4	8
Learning rate	$1\times 10^{-4}$	$1\times 10^{-6}$
Loss function	MSE loss	MSE loss
Training epochs	2000	10,000
Optimizer	Adam^39^	Adam

#### 2.2.2 Spectral unmixing and ROI optimization

Our spectral unmixing is performed using a least squared error (LSE) method as described in our previous studies,^19^ which is also presented in Appendix A. The compensations for light propagation differences of wavelengths are determined based on an experimental measurement of photoacoustic intensity attenuation on *ex vivo* tissue.

**Fig. 5:**
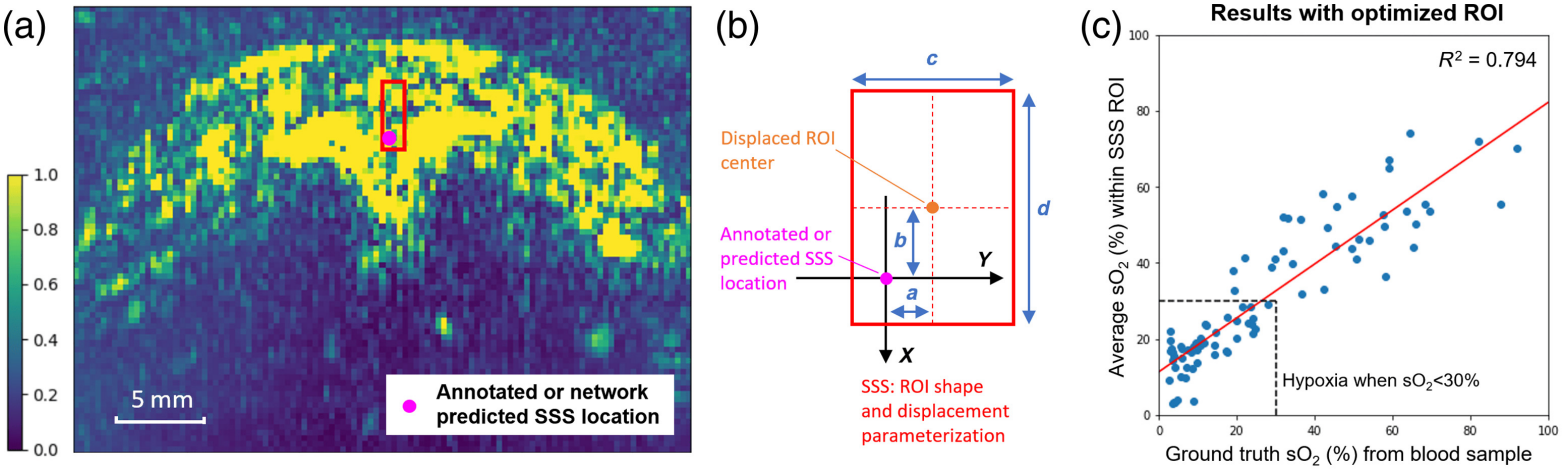
ROI optimization for SSS representation. (a) Annotated/predicted SSS location and the optimized ROI representation overlaid on the original PA image. (b) ROI parameterization for both size and displacement. (c) $O_{2}\mathrm{Sat}$-map-based oxygen level reading compared with blood-sample-based reading using optimized ROI.

Although the conventional per-pixel spectral unmixing can straightforwardly reconstruct the $O_{2}\mathrm{Sat}$ maps within the imaging FOV, no clinical implication can be generated until we specify the SSS ROI for representing brain oxygenation status. In step 1 of our $O_{2}{\mathrm{Sat}}_{\mathrm{ss}}$ estimation workflow [Fig. 3(a)], the location of SSS is detected by SSSLocNet. However, the network-predicted location of the SSS, as well as the expert annotated location of the SSS, is only a point on the image, and the actual region that corresponds to the SSS should be more than a single pixel with an undetermined size and displacement from the annotated/predicted SSS location. Therefore, to best represent the SSS with a rectangular-shaped ROI, we have parameterized its shape and displacement, as shown in Fig. 5(b).

A grid-search optimization is carried out to identify the optimal ROI parameters so that the parameterization best fits the acquired blood sample readings. The median diameter of SSS in fetal newborns is 3.6 mm according to the literature^40^ and is assumed to be similar for piglets. Therefore, to account for the variations of different subject anatomies and test all plausible configurations, the search space for the ROI center displacement (a and b) is set as [–5, 5] pixels ([–1.5 mm, 1.5 mm]), and the search space of ROI shape size parameters (c and d) and is set as [2, 20] pixels ([0.6 mm, 6 mm]). One additional search dimension is the kernel size σ of the Gaussian filter applied on the $O_{2}\mathrm{Sat}$ maps—because the spectral unmixing can be noisy with large variations among neighboring pixels, Gaussian smoothing can help improve the robustness of the estimation result. During the grid search optimization, the cost function is set as the mean squared error (MSE) between the estimated and ground truth readings (percentage of oxygenation). One additional search criterion is that the sensitivity and specificity of classifying hypoxia ($O_{2}{\mathrm{Sat}}_{\mathrm{ss}}<30\%$) versus normoxia ($O_{2}{\mathrm{Sat}}_{\mathrm{ss}}\geq 30\%$) should both be >90%. Because blood sample readings can be biased toward lower $O_{2}\mathrm{Sat}$ readings due to the inaccuracies from the blood gas machine (more discussion in Sec. 4), we do not solely rely on MSE for optimization with blood sample readings and the classification-based criterion can help regularize the ROI parameterization.

The optimization results are shown in Table 2. The positive values for parameters *a* and *b* represent the amount of displacement of ROI following the same axis direction, as shown in Fig. 5(b), and values for parameters *c* and *d* represent the ROI edge lengths. Given the result, the highlighted setting with minimum MSE value was chosen and applied to all the following experiments. This optimized ROI is an elongated shape, as shown in Fig. 5(b), which has its biological interpretation: (1) it focuses on the brain center line, so it will not take the irrelevant signals from the minor vessels on the side, and (2) SSS is often conformed to the brain curvature like an inverted equilateral triangle, which may also have longer dimension in depth direction, as typically and diagrammatically demonstrated for the SSS anatomy.^41^ With this setting, we can achieve a linear regression result between the ground truth and estimated $O_{2}{\mathrm{Sat}}_{\mathrm{ss}}$ values with an *R*-squared value (also known as coefficient of determination or $R^{2}$) of 0.794, *y*-axis intercept of 11.147, and slope of 0.709, as shown in Fig. 5(b).

**Table 2:** ROI grid search optimization result given the expert annotation of SSS location.

Gaussian kernel σ (pixels)	Optimal MSE with GT	Hypoxia sensitivity (%)	Hypoxia specificity (%)	ROI parameter *d* (pixels)	ROI parameter *c* (pixels)	ROI parameter *b* (pixels)	ROI parameter *a* (pixels)
1	**120.39**	**90.74**	**100**	**11**	**4**	**−4**	**1**
2	124.01	90.74	100	9	2	−4	1
3	130.97	90.74	96.67	3	2	−4	1
4	140.49	90.74	93.33	2	2	−5	1

Pixel spacing for the PA image data in both *x*- and *y*-directions is 0.3mm. For the ROI parameters, *d*, optimal ROI edge length in the *x*-axis; *c*, optimal ROI edge length in the *y*-axis; *b*, optimal ROI center displacement in the *x*-axis; *a*, optimal ROI center displacement in the *y*-axis. “Optimal” represents the optimization result from each Gaussian kernel size σ. Note: bold face here means it’s achieving minimal error given the ROI configuration of the row.

### 2.3 Comparative End-to-End Learning-Based Oxygenation Level Estimation Workflow

Using SSS ROI detection as an intermediate step for $O_{2}{\mathrm{Sat}}_{\mathrm{ss}}$ estimation workflow can be more explainable as the clinician is able to visually check the SSS localization results. However, even with expert annotations, the $O_{2}{\mathrm{Sat}}_{\mathrm{ss}}$ estimations report an *R*-squared value of 0.794 when compared against the ground truth, which is promising but still has room for further improvement. Therefore, we decided to also evaluate an alternative approach where $O_{2}{\mathrm{Sat}}_{\mathrm{ss}}$ estimations are directly produced by a neural network with end-to-end learning, using the spectrum images and/or $O_{2}\mathrm{Sat}$ maps as input to utilize the available information from the entire field of view.

**Fig. 6:**
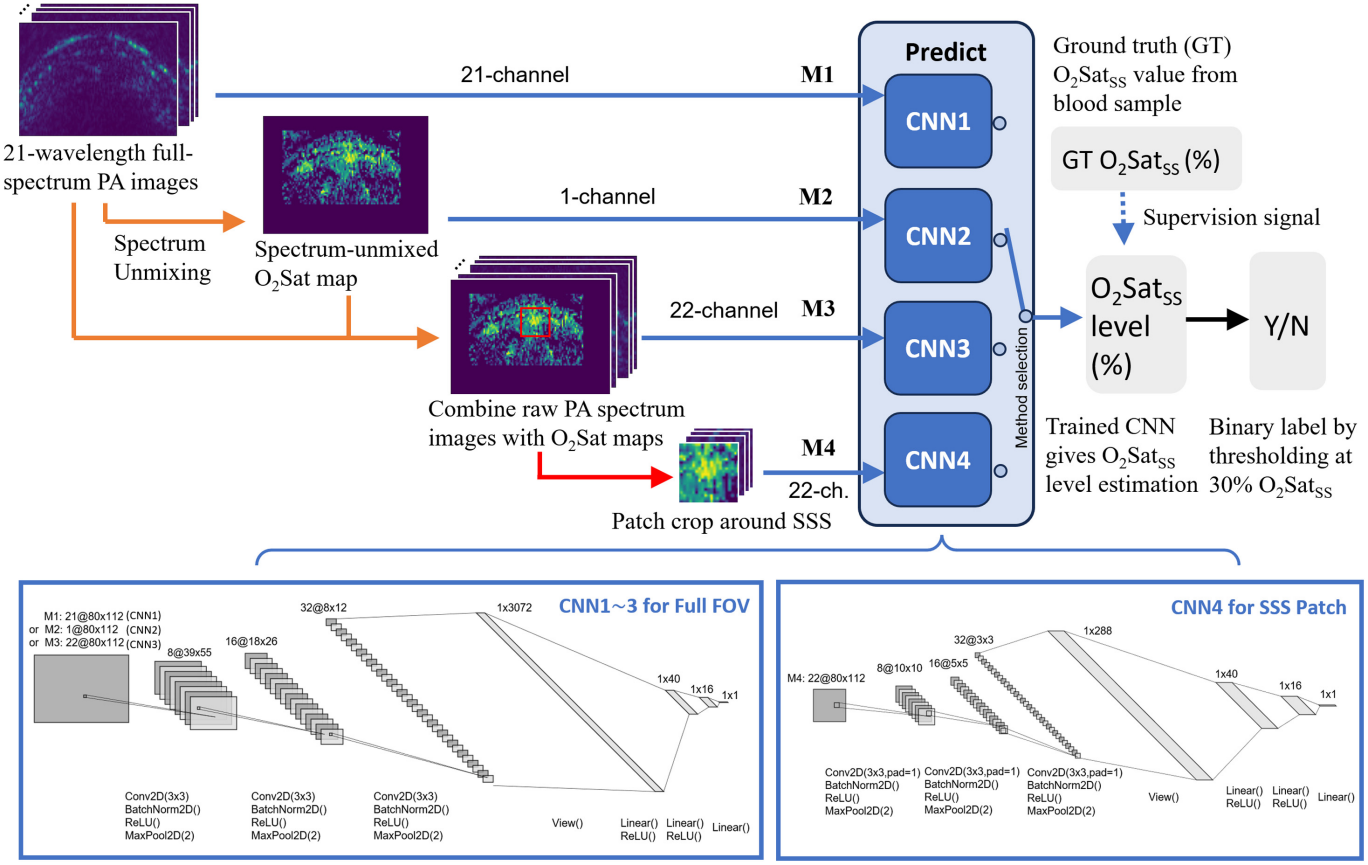
End-to-end learning workflow for $O_{2}\mathrm{Sat}$ estimation. M1 to M4 corresponds to methods with different inputs and different CNNs. The CNN model for M1 to M3 remains the same except for the input, and a smaller CNN model for M4 is used due to the smaller input size.

The alternative end-to-end learning workflow is shown in Fig. 6. To avoid overfitting the training data, a smaller version of the LeNet-type^42^ CNN is used to take in the raw PA spectrum images (or the $O_{2}\mathrm{Sat}$ maps) and generate predictions of $O_{2}{\mathrm{Sat}}_{\mathrm{ss}}$ values. Based on the different input configurations, four methods have been implemented. The first method (M1) takes in the raw PA spectrum images (21-channel). Because the spectrum information (relative intensity between images of different wavelengths) needs to be retained for the network to make predictions for the $O_{2}{\mathrm{Sat}}_{\mathrm{ss}}$ value, no dynamic range rectification was applied, and the raw PA images were only min–max normalized between [0,1] as the network input. The same normalization was applied to all the following methods involving raw PA spectrum images. The second method (M2) takes in the spectral unmixed $O_{2}\mathrm{Sat}$ maps (1 channel) as input. Because the PA signal compensation for spectral unmixing is optimized for imaging depth at the level of SSS, only the region of size 50 × 80 pixels around SSS is spectral unmixed, whereas the remaining area is masked with zeros. The third method (M3) combines both the raw PA spectrum images and the $O_{2}\mathrm{Sat}$ maps to constitute a 22-channel input. In the end, the fourth method (M4) crops a 20 × 20 pixels image patch around SSS from the 22-channel input images of M3, and a smaller CNN is trained with the patch input to estimate the $O_{2}{\mathrm{Sat}}_{\mathrm{ss}}$ level. The 20 × 20 pixels patch (6 × 6 mm) is considered to focus the network on the SSS information (pixels within the optimized ROI region) while incorporating the context information of only the neighboring pixels (the remaining pixels within the patch). To make M4 fully autonomous, SSS locations can be assumed to be generated by algorithm step 1 in Fig. 3.

During the training time, the network training hyperparameters are set according to Table 1 ($O_{2}\mathrm{Sat}$ PredNet). Geometric data augmentations, including horizontal flip of the image, grid distortion, random image shifting, rotation, and scaling, were applied to all input formats. Because ∼70% of the data points are hypoxic ($O_{2}{\mathrm{Sat}}_{\mathrm{ss}}<30\%$), to overcome the challenge of data imbalance, data points are grouped into hypoxia and normal sets. During training, the training batch will randomly draw from the two groups with equal probability.

## 3 Experiments and Results

It is crucial to evaluate the generalizability of the proposed methods on unseen subjects or anatomy. Therefore, leave-one-out cross-validation was implemented for all the following experiments: in the first iteration, out of the 10 piglet subjects, data points from nine subjects were used for training and 1 for testing. In the next iteration, we choose a different piglet subject for testing and the remaining nine subjects for training. By iterating this process 10 times with different testing subjects, we can obtain the method performance metric over the entire dataset, such that the trained algorithm is always tested on the piglet subject unseen by the trained network.

### 3.1 Superior Sagittal Sinus Localization Accuracy

In the two-step $O_{2}{\mathrm{Sat}}_{\mathrm{ss}}$ estimation workflow, spectral unmixing to obtain $O_{2}\mathrm{Sat}$ maps from raw PA spectrum images is a model-based approach that is agnostic of the testing subjects, whereas the localization of SSS landmarks is purely data-driven using a trained U-Net. Therefore, it is important to examine the generalizability of the trained U-Net to unseen anatomy. Given the cross-validation experiments, we can obtain the predictions for the SSS landmark locations for all 84 data points and compare them against our expert annotations of SSS location.

**Fig. 7:**
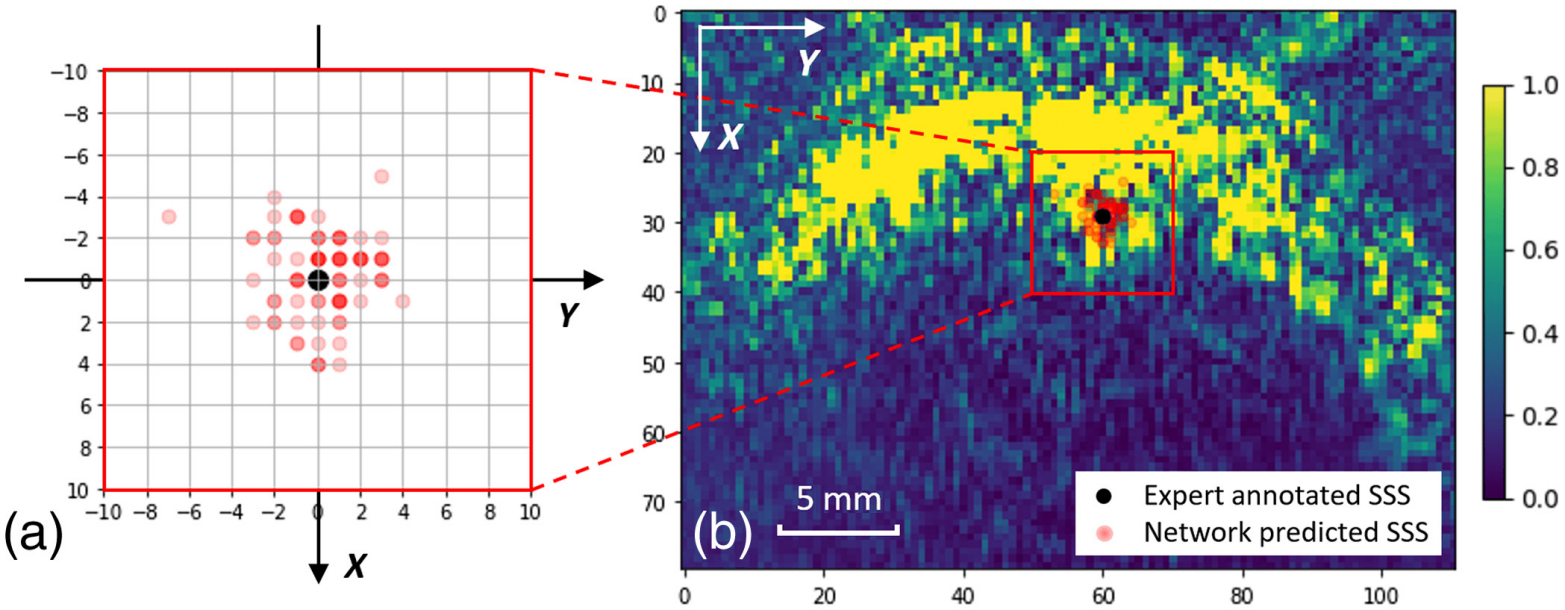
SSS localization error distribution. (a) Zoom-in view of the SSS location prediction compared with the expert annotation for all 84 data points. (b) The predictions and expert annotation overlaid on one example PA image for scale comparison (expert annotation location is from the same example image).

We subtracted the coordinates of all expert annotated SSS locations from the corresponding predicted SSS locations so that we can obtain a relative SSS prediction map, as shown in Fig. 7(a). Because of pixel discretization, predicted SSS locations for different data points may overlap. We used transparency of the red dots to represent sample occurrence such that opaquer means more dots overlapped. We can observe that most predicted SSS locations are within four pixels (pixel spacing 0.3 mm) from the expert annotated locations. The quantitative result shows that the mean and standard deviation for the SSS location prediction are 1.57±1.92 pixels (0.47±0.58  mm) and 1.40±1.82 pixels (0.42±0.55  mm) away relative to the ground truth location in the *X* and *Y* axes, respectively. A visual illustration of the SSS location prediction results is shown in Fig. 7(b): the relative SSS prediction map is overlaid on top of an example PA image, such that the origin of the relative prediction map is placed at the ground truth SSS location of the example PA image. Based on the direct scale comparison, it can be seen that most of the predictions are located at the “dent” of the “M”-shaped brain curvature where the SSS should theoretically reside, i.e., on the midline above the falx cerebri. Therefore, this result demonstrates the robustness of our SSS landmark localization step on unseen piglet subjects.

### 3.2 Oxygen Level Estimation Performance with Different Workflows

**Fig. 8:**
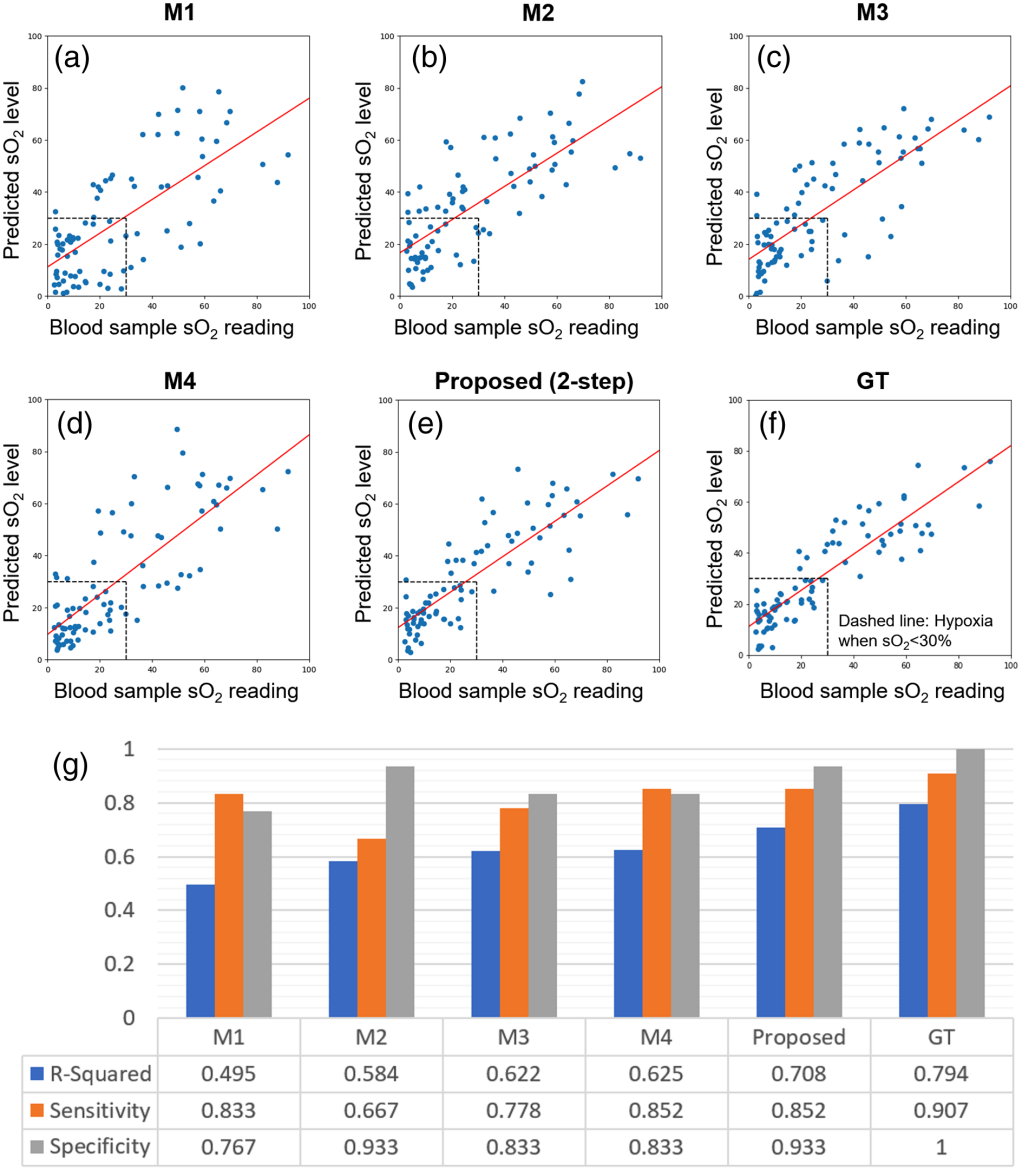
$O_{2}{\mathrm{Sat}}_{\mathrm{ss}}$ prediction performance quantification with cross-validation experiments over the entire dataset. (a)–(f) Experimental results with different workflows: The proposed method stands for the two-step SSS-localization/spectral unmixing approach, M1 to M4 represents the end-to-end learning approach with different inputs and GT is the expert SSS annotation with ROI spectral unmixing. (g) Classification and regression metrics for different $O_{2}{\mathrm{Sat}}_{\mathrm{ss}}$ prediction workflows.

Given the predicted SSS location, an optimized ROI patch is cropped from the spectral unmixed $O_{2}\mathrm{Sat}$ map, with parameters obtained from Sec. 2.2.2. The average intensity value of the cropped patch is used as the final $O_{2}\mathrm{Sat}$ prediction value of our proposed two-step estimation workflow. The experimental result for this proposed two-step workflow is shown in Fig. 8(e). We have also evaluated the end-to-end learning-based workflows presented in Sec. 2.3, and the results for methods M1 to M4 in Fig. 6 are shown in Figs. 8(a)–8(d), respectively. Note that for M4, the 20 × 20 pixels patch was cropped around the expert-annotated SSS location as input, instead of using the results from SSSLocNet. This allows us to quantify the $O_{2}{\mathrm{Sat}}_{\mathrm{ss}}$ prediction capability by itself when perfect SSS localization is assumed. In the end, the performance of the two-step $O_{2}{\mathrm{Sat}}_{\mathrm{ss}}$ estimation with expert annotation (ground truth) is shown in Fig. 8(f).

With the results of $O_{2}{\mathrm{Sat}}_{\mathrm{ss}}$ prediction in Figs. 8(a)–8(f) compared against blood sample readings, we can perform linear least-square regression for the two measurements and obtain the *R*-squared value for each of the examined workflows, which provides a measure of how well the predictor variable (ground-truth $O_{2}{\mathrm{Sat}}_{\mathrm{ss}}$ from blood gases) can explain the variation in the response variable (predicted $O_{2}{\mathrm{Sat}}_{\mathrm{ss}}$). In other words, it is a measure of goodness of fit, but different from MSE, it is conveniently scaled between 0 and 1 and can be directly compared with other works of literature. Therefore, it is chosen as the key performance index for the workflows examined. In addition, the oxygen level at 30% is set as the classification boundary between hypoxia versus normoxia cases. This boundary is based on the assumptions that fetal preductal arterial $O_{2}\mathrm{Sat}$ in a healthy fetus is expected to be 65% to 75% and that the healthy fetal brain extracts 30% to 40% of delivered O_2_, resulting in an estimated normal $O_{2}{\mathrm{Sat}}_{\mathrm{ss}}$ in the range of 39% to 53% for a healthy fetus.^43^^,^^44^ As a result, the sensitivity and specificity of predicting hypoxia cases via the use of different workflows can be computed. Given the performance of these metrics as shown in Fig. 8(g), we can observe that the two-step SSS localization-based approach outperforms the end-to-end learning workflows and is closest to the performance of human expert labeling.

### 3.3 Prediction Confidence Quantification with Monte Carlo Dropout

For translational implementation, instead of only showing the clinician a binary indicator of fetal brain hypoxia, it is also highly desirable to know the confidence of the oxygen level estimation made by the system. Therefore, in this study, we also present a way to quantify the prediction uncertainty ε via Monte Carlo dropout (MCD).

#### 3.3.1 Model uncertainty

**Fig. 9:**
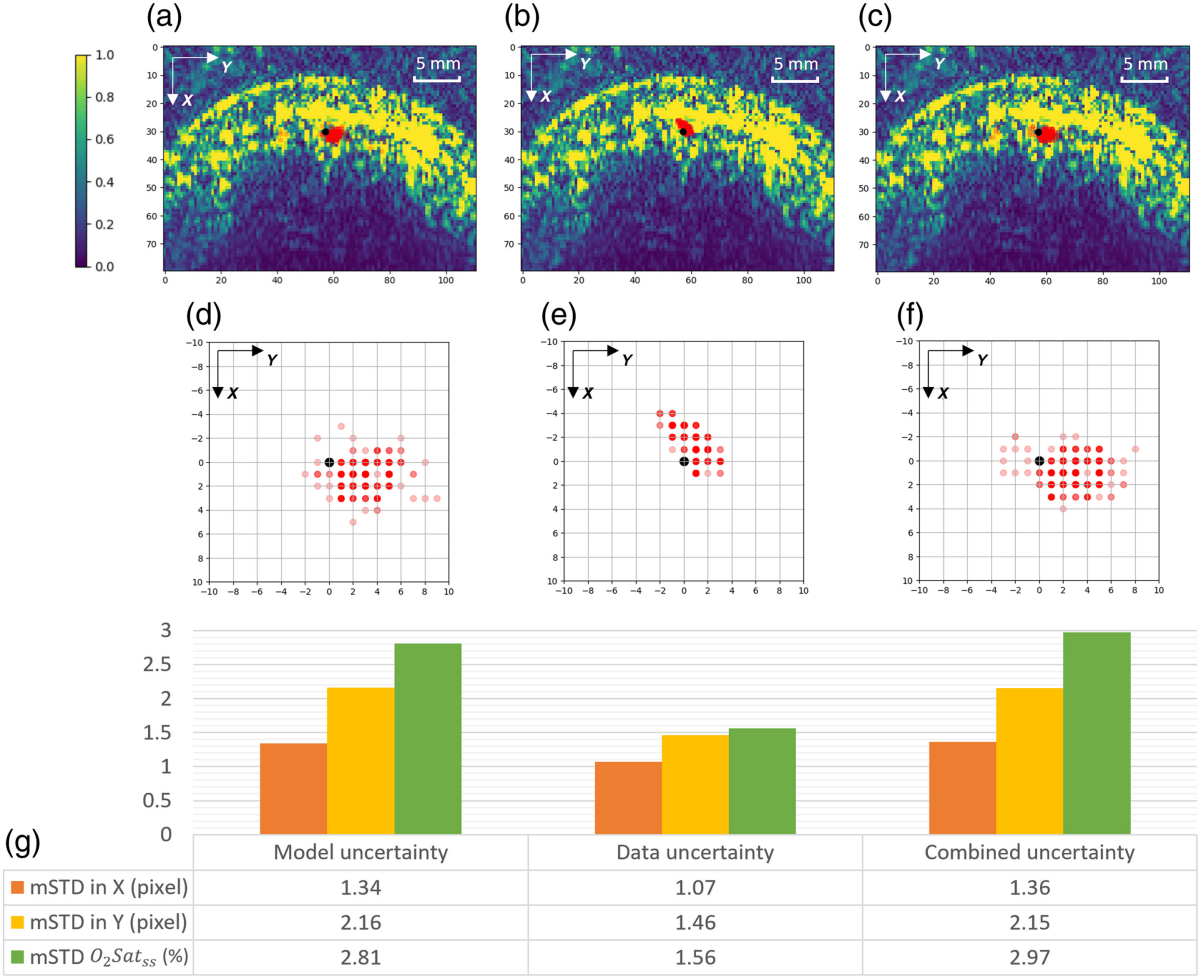
Uncertainty quantification with Monte Carlo dropout (MCD). (a), (d) SSS localization distribution example with model-MCD overlaid on the input raw PA image and its zoom-in view. The opaqueness of the red dots indicates multiple occurrences at the same locations. (b), (e) The distribution example with data MCD. (c), (f) The distribution example with combined (model + data) MCD. (g) Mean STD (mSTD) for different types of uncertainties over the entire dataset, and sO2 stands for the $O_{2}{\mathrm{Sat}}_{\mathrm{ss}}$ prediction.

The U-Net in our proposed method contains dropout layers after each max-pooling or up-sampling operation (as shown in Fig. 3), and we can in fact run inferences with these dropout layers being turned on during the test time. With multiple runs, each SSS location prediction can vary due to the change of network structures even with the same image input, which leads to different predictions of $O_{2}{\mathrm{Sat}}_{\mathrm{ss}}$. Therefore, we can compute the standard deviation of the predicted SSS coordinates and the resulting predicted $O_{2}{\mathrm{Sat}}_{\mathrm{ss}}$ values as the model uncertainty. In this experiment, we ran 400 times for each data point in our dataset. An example is shown in Figs. 9(a) and 9(d), where it shows the distribution of the SSS localization with MCD. A standard deviation (STD) can be computed for this data point and will be used as the uncertainty value ϵ for this data point. For example, if ϵ=20%, the model is reporting the $O_{2}{\mathrm{Sat}}_{\mathrm{ss}}$ with ±20% STD, and we define the confidence score β=1−2·ϵ, which is 60% confidence for this prediction. Furthermore, when we compute the mean of standard deviations (mSTD) of predictions over all data points, it represents the average uncertainty for our method on the entire dataset. As shown in Fig. 9(g), the model variation gives us $O_{2}{\mathrm{Sat}}_{\mathrm{ss}}$ prediction mSTD of 2.81%, and we have average uncertainty ϵ=2.81% and average confidence β=94.3%. This result indicates that even with dramatic modifications (50%) of network connections in dropout layers, the oxygenation level predictions remain stable.

#### 3.3.2 Data uncertainty

The raw PA spectrum has 21 wavelengths, and only five wavelengths were randomly chosen as input during training time (Sec. 2.2.1) as a data augmentation method. If we choose to keep the partial-spectrum input during test time, we are essentially running the MCD with data variation. Therefore, we can quantify the uncertainty of predictions attributed to the data. In our experiment, we ran 400 times for each data point in our dataset. An example is shown in Figs. 9(b) and 9(e), and the data variations give us $O_{2}{\mathrm{Sat}}_{\mathrm{ss}}$ average uncertainty ϵ of 1.56%, thus an average confidence score β of 96.88%.

#### 3.3.3 Combined uncertainty

We evaluated the model and data uncertainties individually, but it is also beneficial to quantify the uncertainty when running MCD on the model and data together. Therefore, in this experiment, we set 20 model variations and 20 data variations, which multiply into 400 different configurations for one data point. An example of SSS prediction variations on a single data point is shown in Figs. 9(c) and 9(f), and the mSTD of $O_{2}{\mathrm{Sat}}_{\mathrm{ss}}$ predictions over the entire dataset is shown in Fig. 9(g). We noticed that the average combined uncertainty ϵ for predicted $O_{2}{\mathrm{Sat}}_{\mathrm{ss}}$ is 2.97%, thus an average confidence score β of 94.06%.

## 4 Discussions

As shown in Fig. 8(g), our proposed two-step landmark detection-based approach outperforms all the end-to-end learning-based methods (M1 to M4) in all metrics, with an *R*-squared value of 0.708, an 85.2% sensitivity, and 93.3% specificity. With the combined (model/data) MCD uncertainty quantification, we observed a stable performance with ±1.36 and ±2.15 pixels (±0.41 and ±0.65  mm) mSTD in *X* and *Y* directions for SSS localization, respectively, and combined uncertainty value ϵ of 2.97% for final $O_{2}{\mathrm{Sat}}_{\mathrm{ss}}$ prediction. Given these results, several insights are identified in the following discussion.

### 4.1 SSS Matters

In addition to the superior performance of our proposed two-step method, we also notice that the patch-based end-to-end learning method (M4 in Fig. 6) outperforms the other end-to-end learning methods M1 to M3. This result indicates that the region of SSS contains the information of the highest correlation with the ground-truth blood gas measurements. It also suggests that the signal responses from minor blood vessels from other brain structures can be noisy and misleading as they can be inaccurate due to unoptimized PA attenuation compensation (the PA signal attenuation compensation is determined based on the typical imaging depth of SSS). Therefore, the end-to-end learning networks cannot correctly extract the key information if provided with information from the entire FOV without providing a large amount of training data. As a result, the final $O_{2}{\mathrm{Sat}}_{\mathrm{ss}}$ from our two-step algorithm has the advantage of taking only the average spectral unmixed ${\mathrm{sO}}_{2}$ value over the most informative SSS region.

### 4.2 Ground Truth SSS Annotation Relies on the Anatomical Knowledge

The SSS structure can hardly be seen in the transcranial B-Mode images, but the photoacoustic signals illustrate a context-rich brain image, as shown in Fig. 1(b). Therefore, the SSS locations can be identified via virtually mapping the image features to the actual brain structures by experts with knowledge of both photoacoustic imaging and porcine brain anatomy. In this work, the annotation used for every data sample is the consensus result of two experts experienced with PA imaging and brain anatomy.

### 4.3 Interpretable Two-Step Approach

The only nonmodel-based algorithmic part in our proposed $O_{2}{\mathrm{Sat}}_{\mathrm{ss}}$ estimation workflow is the SSS landmark detection, which generates the intermediate output of the SSS localization heatmap that can be directly examined by human experts. In the case when high uncertainty/low confidence is reported with MCD runs during continuous hypoxia monitoring, experts can view the network output heatmap directly and compare it against the PA images for confirmation, e.g., checking whether the predicted SSS location is on the midline above the falx cerebri. This presents another advantage of our proposed method over the end-to-end learning-based methods of which the prediction is less interpretable.

### 4.4 Correct Plane of Imaging Is Necessary

The U-Net–based SSS landmark detection is enabled by training with the consistent anatomical patterns, i.e., the imaging plane is almost orthogonal to the brain surface and contains the SSS. Otherwise, the network cannot correctly identify the correct ROI to compute the $O_{2}{\mathrm{Sat}}_{\mathrm{ss}}$ prediction from the spectral unmixing map. For this purpose, 5 out of 15 subjects in our original *in vivo* piglet study were discarded due to less satisfactory data quality that either SSS is missing from FOV, or probe is tilted with an angle that shows different anatomical structures and feature shapes. A detailed description of our data curation process is presented in Appendix B. For clinical translation, a cost-efficient PA imaging system, either handheld or wearable form factor, should be guided to be affixed to ensure correct imaging plane orientation when being deployed to patients. In addition, for any 3D wearable PA imaging device that generates volumetric signals, with standard plane detection techniques,^45^ we can also extract the correct plane of imaging from a 3D volume and easily integrate with the two-step $O_{2}{\mathrm{Sat}}_{\mathrm{ss}}$ estimation workflow proposed in this work.

### 4.5 Blood Sample Reading Is the Best But Not Perfect Ground Truth

For *in vivo* experiments, it is crucial to know the ground truth for the measurements taken. In our setup, blood samples are drawn from the brain vessel after each spectrum PA imaging measurement. However, it is important to realize that our blood gas machine is for clinical use with human hemoglobin (Hb) in which a spectral fit is made to account for COHb, metHb, and O2Hb. Piglet Hb may have a different spectral fit for these three states of Hb. In addition, when O2Hb saturation decreases during hypoxia, the amount of COHb and metHb does not decrease, so their contribution to the fractional error in estimating O_2_Hb could become larger on a relative basis. Therefore, the blood gas machine could have some error, and we see in Figs. 8(f) and 8(g) that even with expert SSS location annotation, the $O_{2}{\mathrm{Sat}}_{\mathrm{ss}}$ estimation is not perfect and has an obvious *y*-intercept value at 12.3% after linear least square regression. This imperfection in data sets an upper bound to any method proposed, and we expect that a better performance can be achieved by the same methods with a cleaner dataset and more accurate measurement of the ground truths.

### 4.6 Model Variation Is the Major Cause of Uncertainty

From the experimental results shown in Fig. 9. We observe that the model uncertainty metrics are on par with the combined uncertainties, whereas the data-induced uncertainty is secondary. This demonstrates that with MCD applied to the model, a larger variation can be observed on SSS localization than varying the input spectrum selection from PA images. Therefore, in the implementation of real-time uncertainty quantification, we may elect to run MCD on model variations only for faster computation.

### 4.7 $O_{2}{\mathrm{Sat}}_{\mathrm{ss}}$ Is Used to Detect Global Hypoxia Rather than Local Hypoxia-Ischemia

In this work, our animal model was designed to introduce global hypoxia, rather than local hypoxia-ischemia. Given that our algorithms work with a large field-of-view over the brain coronal plane, we consider identifying the localized hypoxia-ischemia region, instead of identifying SSS location, as an immediate next step. This future work will give a more spatially registered analysis of sagittal sinus and other brain circuitries to understand the overall neurovascular physiology in the patients. Furthermore, it is worth noting that when local hypoxia-ischemia occurs, the hypoxic tissue will likely increase the regional cerebral blood flow (vasodilation) and increase its oxygen extraction fraction, meaning it is pulling more oxygen out of the passing blood.^46^ Therefore, there can still be a nontrivial correlation between the local oxygenation level and $O_{2}Sat_{ss}$, which can be detected autonomously from the methods in this work.

## 5 Conclusion and Future Works

In this work, we presented a fully automated fetal hypoxia monitoring workflow based on spectral photoacoustic imaging and evaluated it with *in vivo* piglet experimental data. A two-step approach was proposed to estimate the $O_{2}{\mathrm{Sat}}_{\mathrm{ss}}$: a U-Net (SSSLocNet) is trained to detect the key anatomical landmark SSS, and an optimized ROI is cropped around the detected SSS location within the spectral unmixed $O_{2}{\mathrm{Sat}}_{\mathrm{ss}}$ map, of which the average intensity value is the final $O_{2}{\mathrm{Sat}}_{\mathrm{ss}}$ estimation.

With leave-one-subject-out cross-validation experiments, we showed that our proposed method outperforms the end-to-end learning-based methods and achieves an R-squared value of 0.708 when compared against the blood sample readings. In addition, with 30% chosen as the threshold for brain hypoxia alarm, an 85.2% sensitivity and 93.3% specificity are achieved. We have also estimated the overall uncertainty for the proposed workflow via Monte Carlo dropout and showed the mSTD for SSS localization is 0.41 and 0.65 mm in *X* and *Y* directions, respectively, and the mSTD for $O_{2}{\mathrm{Sat}}_{\mathrm{ss}}$ prediction is 2.97% in 400 runs with combined (model/data) MCD.

For future translational development, two technology roadmaps can be independently pursued for intrapartum and postpartum monitoring. During the intrapartum phase, we envision that an endovaginal light delivery system can be affixed to the cervix and illuminate the fetal brain,^47^ with volumetric photoacoustic signals being received by an external wearable scanner^48^ and processed to extract the coronal plane of the brain^45^ as the input to the algorithm proposed in this work. On the other hand, during the postpartum phase, a linear PA probe or a wearable system may be attached to the fetal head to monitor the SSS directly (e.g., a scanner being placed at the anterior fontanelle, which is on top of the SSS for easier access.), and the proposed algorithmic workflow will then generate $O_{2}{\mathrm{Sat}}_{\mathrm{ss}}$ readings for continuous brain hypoxia monitoring. These envisioned clinical implementations using a cost-effective 1-D PA imaging probe or wearable system delineates the minimal requirements for PA-based brain monitoring to identify at-risk patients and autonomous clinical decision-making for follow-up treatment and interventions.

For the other future research directions, we plan to evaluate the low-energy LED-based light source for $O_{2}{\mathrm{Sat}}_{\mathrm{ss}}$ estimation, which ultimately can be integrated into a fully autonomous monitoring system for antepartum and intrapartum fetal brain hypoxia detection, as described in the translational development plan. In addition, the two-step workflow can easily incorporate more advanced spectral unmixing methods such as LSD^27^ to generate more accurate $O_{2}Sat$ maps. With the envisioned cost-efficient PA imaging system, the two-step $O_{2}{\mathrm{Sat}}_{\mathrm{ss}}$ estimation workflow presented in this work can provide an easy-to-use noninvasive $O_{2}{\mathrm{Sat}}_{\mathrm{ss}}$ monitoring system with minimum human intervention.

## 6 Appendix A: Least Square-Based Spectral Unmixing

As shown in Fig. 2, oxygen saturation ($O_{2}\mathrm{Sat}$) maps are the straightforward representation of the oxygenation status as these maps are constructed from per-pixel oxygen level estimations. The per-pixel $O_{2}\mathrm{Sat}$ estimations are generated via spectral unmixing: oxyhemoglobin has known spectrophotometric absorbance, i.e., the experimentally calibrated ground truth photoacoustic signal response ($S_{\mathrm{GT}}$) for different optical wavelengths (λ) and different concentrations ($O_{2}\mathrm{Sat}$) of oxyhemoglobin. Therefore, by solving the least square optimization problem below, we can obtain the estimated oxyhemoglobin concentration, i.e., the oxygen saturation, at each pixel:

$$O_{2}\mathrm{Sat}=\underset{O_{2}\overset{˜}{\mathrm{Sat}}}{\arg\;\min}\frac{1}{N_{\lambda }}\underset{\lambda }{\sum }[S_{\text{meas}}(\lambda ,O_{2}\overset{˜}{\mathrm{Sat}})-S_{\mathrm{GT}}(\lambda ,O_{2}\overset{˜}{\mathrm{Sat}}){]}^{2},$$ (1)

where $N_{\lambda }$ is the number of wavelengths used, i.e., 21. $S_{\text{meas}}(\lambda ,O_{2}\overset{˜}{\mathrm{Sat}})$ is the measured photoacoustic signal response for wavelength λ and an assumed oxyhemoglobin concentration $O_{2}\overset{˜}{\mathrm{Sat}}$. $S_{\mathrm{GT}}(\lambda ,O_{2}\overset{˜}{\mathrm{Sat}})$ is the known spectrophotometric reference stored in a table. The $S_{\text{meas}}$ values are compensated for photoacoustic attenuation at each wavelength individually, where the amount of signal compensation is determined based on an experimental measurement of photoacoustic intensity attenuation on *ex vivo* tissue of the same depth as skin-to-SSS.^19^

## 7 Appendix B: Data Curation

It can be a challenging task to maintain a consistent experimental environment for *in vivo* animal experiments. Therefore, we have set up three general data curation criteria for making a structured dataset that can produce more meaningful algorithm evaluations.

**Fig. 10:**
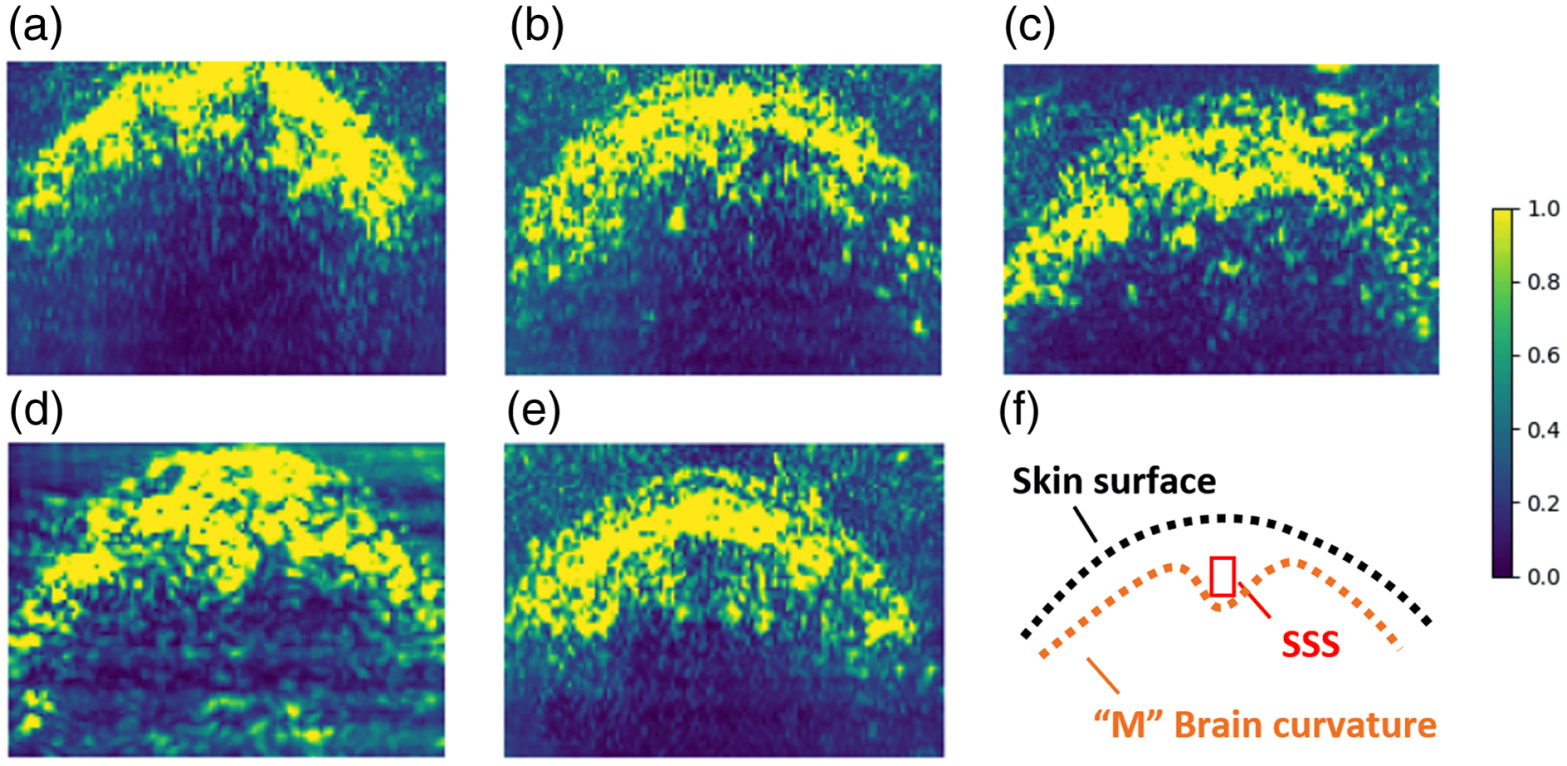
Excluded piglet subjects shown with rectified dynamic range for image plotting. When the PA transducer is placed correctly, i.e., perpendicular to the skull surface above the SSS region, the anatomical structure will follow a general pattern as shown in the bottom right figure with “M” shape brain curvature. (a)–(e) corresponds to the acquired SSS coronal plane PA images of the five excluded piglet subjects A to E, respectively. (f) is the simplified sketch for the brain anatomical structure in coronal view.

1. The entire brain and skull structure are within the imaging FOV. The PA transducer is not attached to the animal and the relative position of the probe and piglet can change due to experimental operations. Therefore, the skull and brain may partly move outside of the imaging FOV, in which case we decide to exclude the data for data consistency. In real-world implementations, the transducer will either be held by the clinician or attached as a wearable device, so we do not expect the target anatomical structure move out of FOV.
2. No data point has abnormal blood sample $O_{2}{\mathrm{Sat}}_{\mathrm{ss}}$ readings. For each piglet subject, we always start with normal (healthy) $O_{2}{\mathrm{Sat}}_{\mathrm{ss}}$ level PA imaging and then gradually reduce the oxygen supply to create the brain hypoxia condition. Therefore, we should always expect to see the $O_{2}{\mathrm{Sat}}_{\mathrm{ss}}$ level being >30% in the first few samples. When the first few data points of a piglet subject show blood sample readings <30%, we suspect there can be data errors due to many possible reasons. For example, the catheter moves out from the SSS and draws blood samples out of the target, or the blood gas reading machine is not clean enough for the day of the experiment and contaminates the blood sample for the piglet subject.
3. The PA transducer is placed perpendicularly to the skull surface above the SSS region. One requirement of our proposed landmark detection-based algorithm is that the imaging FOV shows the target anatomical patterns. To ensure that the “M” brain curvature [as shown in Fig. 10(f)] is clearly visible, the PA transducer should be placed perpendicular to the skull surface above the SSS region. During the animal experiments, the transducer is held by a clamp and not affixed to the piglet head, so it is possible that the imaging plane tilts away from the SSS coronal plane, as shown by an example in Fig. 10(e).

Following these criteria, we will list the reasons for excluding the five piglet subjects below. We denote the five excluded piglet subjects as A to E, as shown in Figs. 10(a)–10(e).

**Fig. 11:**
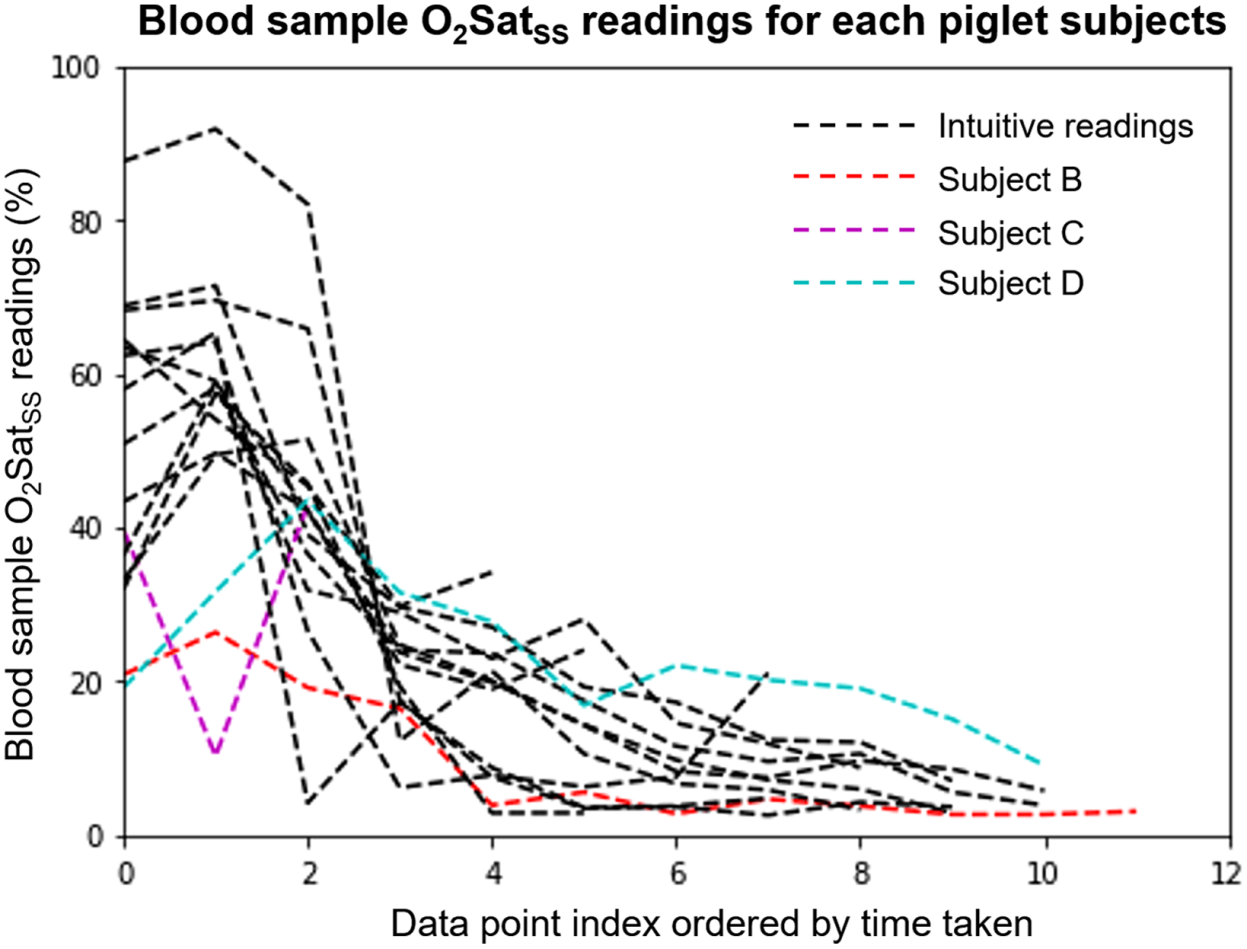
Excluded piglet subjects with abnormal ground truth $O_{2}{\mathrm{Sat}}_{\mathrm{ss}}$ readings when compared with other piglet subjects in the dataset.

1. Piglet A: (criteria 1 and 3) The top part of the skull surface is not visible in the FOV. The PA transducer is also tilting away from the SSS coronal plane and has no clear “M” brain feature.
2. Piglet B: (criterion 2) As shown in Fig. 11, the blood samples for the first few data points show hypoxia ($O_{2}{\mathrm{Sat}}_{\mathrm{ss}}<30\%$) readings, which is counterintuitive as we always start the data collection from the healthy condition first.
3. Piglet C: (criterion 2) As shown in Fig. 11, only three data points were collected for this subject, which indicates that the piglet dies early. In addition, the second data point shows hypoxia ($O_{2}{\mathrm{Sat}}_{\mathrm{ss}}<30\%$) reading, so that blood sample reading error may have occurred.
4. Piglet D: (criterion 2) As shown in Fig. 11, the first two data points show hypoxia ($O_{2}{\mathrm{Sat}}_{\mathrm{ss}}<30\%$) blood sample readings, which indicates the possible blood sample reading error.
5. Piglet E: (criterion 3) As shown in Fig. 10(e), the PA transducer is not placed correctly to image the SSS coronal plane above SSS. Therefore, no clear “M” brain feature is shown, so we exclude this subject from our dataset.

From our reasoning above, five piglet subjects containing data points with compromised quality were excluded. Even though a portion of data points in these excluded subjects are possibly still usable, e.g., those hypoxic data points of piglet B at the ending stage of this piglet experiment, we choose to exclude the entire piglet for consistency and accuracy.

## Data Availability

Code for the algorithms is provided at https://github.com/bcjiang/hypodetect The data from the piglet experiment will be available upon request by email.

## References

[1] Lai M.-C. et al., “Perinatal hypoxic-ischemic encephalopathy,” BioMed. Res. Int. 2011, 609813 (2011). 10.1155/2011/609813 PMC3010686 21197402

[2] Graham E. M. et al., “A systematic review of the role of intrapartum hypoxia-ischemia in the causation of neonatal encephalopathy,” Amer. J. Obstet. Gynecol. 199(6), 587–595 (2008). AJOGAH 0002-9378 10.1016/j.ajog.2008.06.094 19084096

[r3] Clark S. L. Hankins G. D., “Temporal and demographic trends in cerebral palsy—fact and fiction,” Amer. J. Obstet. Gynecol. 188(3), 628–633 (2003). AJOGAH 0002-9378 10.1067/mob.2003.204 12634632

[4] Nelson K. B. et al., “Uncertain value of electronic fetal monitoring in predicting cerebral palsy,” New England J. Med. 334(10), 613–619 (1996). NEJMBH 10.1056/NEJM199603073341001 8592523

[5] de Laveaucoupet J. et al., “Fetal magnetic resonance imaging (MRI) of ischemic brain injury,” Prenatal Diagn. 21(9), 729–736 (2001). PRDIDM 1097-0223 10.1002/pd.135 11559908

[6] Counsell S. J. Rutherford M. A., “Magnetic resonance imaging of the newborn brain,” Curr. Paediatr. 12(5), 401–413 (2002). 10.1054/cupe.2002.0318

[7] Lee S. et al., “Pathways for neuroimaging of neonatal stroke,” Pediatr. Neurol. 69, 37–48 (2017). 10.1016/j.pediatrneurol.2016.12.008 28262550

[8] Salas J. et al., “Head ultrasound in neonatal hypoxic-ischemic injury and its mimickers for clinicians: a review of the patterns of injury and the evolution of findings over time,” Neonatology 114(3), 185–197 (2018). 10.1159/000487913 29936499

[9] Lee C. W. Cooper R. J. Austin T., “Diffuse optical tomography to investigate the newborn brain,” Pediatr. Res. 82(3), 376–386 (2017). PEREBL 0031-3998 10.1038/pr.2017.107 28419082

[10] Semyachkina-Glushkovskaya O. V. et al., “Silent vascular catastrophes in the brain in term newborns: strategies for optical imaging,” IEEE J. Sel. Top. Quantum Electron. 22(3), 88–101 (2016). IJSQEN 1077-260X 10.1109/JSTQE.2016.2523982

[11] Wheelock M. D. Culver J. P. Eggebrecht A. T., “High-density diffuse optical tomography for imaging human brain function,” Rev. Sci. Instrum. 90(5), 051101 (2019). RSINAK 0034-6748 10.1063/1.5086809 31153254 PMC6533110

[12] Benni P. B. et al., “A validation method for near-infrared spectroscopy based tissue oximeters for cerebral and somatic tissue oxygen saturation measurements,” J. Clin. Monit. Comput. 32, 269–284 (2018). 10.1007/s10877-017-0015-1 28374103 PMC5838152

[13] Beard P., “Biomedical photoacoustic imaging,” Interface Focus 1(4), 602–631 (2011). 10.1098/rsfs.2011.0028 22866233 PMC3262268

[14] Song H. Song T.-K. Kang J., “High-contrast spectroscopic photoacoustic characterization of thermal tissue ablation in the visible spectrum,” Ultrasonography 42(2), 249–258 (2022). 10.14366/usg.22171 36935599 PMC10071053

[r15] Kang J. et al., “Transcranial photoacoustic imaging of NMDA-evoked focal circuit dynamics in the rat hippocampus,” J. Neural Eng. 17(2), 025001 (2020).1741-2560 10.1088/1741-2552/ab78ca 32084654 PMC7145727

[r16] Wu Y. et al., “System-level optimization in spectroscopic photoacoustic imaging of prostate cancer,” Photoacoustics 27, 100378 (2022). 10.1016/j.pacs.2022.100378 36068804 PMC9441267

[r17] Kang J. et al., “Real-time sentinel lymph node biopsy guidance using combined ultrasound, photoacoustic, fluorescence imaging: in vivo proof-of-principle and validation with nodal obstruction,” Sci. Rep. 7(1), 45008 (2017). SRCEC3 2045-2322 10.1038/srep45008 28327582 PMC5361205

[r18] Cao F. et al., “Photoacoustic imaging in oxygen detection,” Appl. Sci. 7(12), 1262 (2017). 10.3390/app7121262

[r19] Kang J. et al., “Validation of noninvasive photoacoustic measurements of sagittal sinus oxyhemoglobin saturation in hypoxic neonatal piglets,” J. Appl. Physiol. 125(4), 983–989 (2018). 10.1152/japplphysiol.00184.2018 29927734 PMC6335091

[20] Kang J. et al., “Transcranial photoacoustic characterization of neurovascular physiology during early-stage photothrombotic stroke in neonatal piglets in vivo,” J. Neural Eng. 18(6), 065001 (2022).1741-2560 10.1088/1741-2552/ac4596 PMC9112348 34937013

[21] Gröhl J. et al., “Deep learning for biomedical photoacoustic imaging: a review,” Photoacoustics 22, 100241 (2021). 10.1016/j.pacs.2021.100241 33717977 PMC7932894

[22] LeCun Y. Bengio Y. Hinton G., “Deep learning,” Nature 521(7553), 436–444 (2015). 10.1038/nature14539 26017442

[23] Yang C. Gao F., “EDA-Net: dense aggregation of deep and shallow information achieves quantitative photoacoustic blood oxygenation imaging deep in human breast,” Lect. Notes Comput. Sci. 11764, 246–254 (2019). LNCSD9 0302-9743 10.1007/978-3-030-32239-7_28

[r24] Luke G. P. et al., “O-net: a convolutional neural network for quantitative photoacoustic image segmentation and oximetry,” arXiv:1911.01935 (2019).

[r25] Hoffer-Hawlik K. Luke G. P., “absO2luteU-Net: tissue oxygenation calculation using photoacoustic imaging and convolutional neural networks,” Dissertation, Vol. 1 (2019).

[26] Bench C. Hauptmann A. Cox B., “Toward accurate quantitative photoacoustic imaging: learning vascular blood oxygen saturation in three dimensions,” J. Biomed. Opt. 25(8), 085003 (2020). JBOPFO 1083-3668 10.1117/1.JBO.25.8.085003 32840068 PMC7443711

[27] Gröhl J. et al., “Learned spectral decoloring enables photoacoustic oximetry,” Sci. Rep. 11(1), 6565 (2021). SRCEC3 2045-2322 10.1038/s41598-021-83405-8 33753769 PMC7985523

[28] Kirchner T. Frenz M., “Multiple illumination learned spectral decoloring for quantitative optoacoustic oximetry imaging,” J. Biomed. Opt. 26(8), 085001 (2021). JBOPFO 1083-3668 10.1117/1.JBO.26.8.085001 34350736 PMC8336722

[29] Kirchner T. Jaeger M. Frenz M., “Machine learning enabled multiple illumination quantitative optoacoustic oximetry imaging in humans,” Biomed. Opt. Express 13(5), 2655–2667 (2022). BOEICL 2156-7085 10.1364/BOE.455514 35774340 PMC9203099

[30] Letchuman V. Donohoe C., Neuroanatomy, Superior Sagittal Sinus, StatPearls Publishing, Treasure Island, Florida (2023). 31536222

[31] Hariri A. et al., “Functional photoacoustic tomography for neonatal brain imaging: developments and challenges,” Proc. SPIE 10064, 100642Z (2017). PSISDG 0277-786X 10.1117/12.2254861

[r32] Petrov I. et al., “Optoacoustic monitoring of cerebral venous blood oxygenation though intact scalp in large animals,” Opt. Express 20(4), 4159–4167 (2012). OPEXFF 1094-4087 10.1364/OE.20.004159 22418173 PMC3482910

[33] Petrova I. et al., “Noninvasive monitoring of cerebral blood oxygenation in ovine superior sagittal sinus with novel multi-wavelength optoacoustic system,” Opt. Express 17(9), 7285–7294 (2009). OPEXFF 1094-4087 10.1364/OE.17.007285 19399105

[34] Ronneberger O. Fischer P. Brox T., “U-Net: convolutional networks for biomedical image segmentation,” Lect. Notes Comput. Sci. 9351, 234–241 (2015). LNCSD9 0302-9743 10.1007/978-3-319-24574-4_28

[35] Conrad M. S. et al., “Magnetic resonance imaging of the neonatal piglet brain,” Pediatr. Res. 71(2), 179–184 (2012). PEREBL 0031-3998 10.1038/pr.2011.21 22258129

[36] Conrad M. S. Dilger R. N. Johnson R. W., “Brain growth of the domestic pig (*Sus scrofa*) from 2 to 24 weeks of age: a longitudinal MRI study,” Dev. Neurosci. 34(4), 291–298 (2012). 10.1159/000339311 22777003 PMC3646377

[37] Srivastava N. et al., “Dropout: a simple way to prevent neural networks from overfitting,” J. Mach. Learn. Res. 15(1), 1929–1958 (2014).

[38] Buslaev A. et al., “Albumentations: fast and flexible image augmentations,” Information 11(2), 125 (2020). 10.3390/info11020125

[39] Kingma D. P. Ba J., “Adam: a method for stochastic optimization,” arXiv:1412.6980 (2014).

[40] Saliou G. et al., “Decreased superior sagittal sinus diameter and jugular bulb narrowing are associated with poor clinical outcome in vein of Galen arteriovenous malformation,” Amer. J. Neuroradiol. 37(7), 1354–1358 (2016). 10.3174/ajnr.A4697 26915567 PMC7960337

[41] WikiDoc Contributors, “Diagrammatic section of scalp: superior sagittal sinus,” 2025, https://www.wikidoc.org/index.php/Superior_sagittal_sinus (accessed 30 January 2025).

[42] LeCun Y. et al., “Gradient-based learning applied to document recognition,” Proc. IEEE 86(11), 2278–2324 (1998). IEEPAD 0018-9219 10.1109/5.726791

[43] Jones M. D. Jr. et al., “Oxygen delivery to the brain before and after birth,” Science 216(4543), 324–325 (1982). SCIEAS 0036-8075 10.1126/science.6801768 6801768

[44] Koehler R. C., “Regulation of the cerebral circulation during development,” Compr. Physiol. 11(4), 2371 (2021). 10.1002/j.2040-4603.2021.tb00184.x 34558670 PMC9789530

[45] Jiang B. et al., “Standard plane extraction from 3D ultrasound with 6-DoF deep reinforcement learning agent,” in IEEE Int. Ultrason. Symp. (IUS), IEEE, pp. 1–4 (2020). 10.1109/IUS46767.2020.9251555

[46] Wood C. E. Keller-Wood M., “Current paradigms and new perspectives on fetal hypoxia: implications for fetal brain development in late gestation,” Amer. J. Physiol.-Regul. Integr. Comparat. Physiol. 317(1), R1–R13 (2019). 10.1152/ajpregu.00008.2019 PMC6692760 31017808

[47] Kang J. et al., “Wearable ultrasound and photoacoustic device for fetal and/or labor monitoring,” US Patent App. 18/543,277 (2024).

[48] Xu K. et al., “Autoinfocus, a new paradigm for ultrasound-guided spine intervention: a multi-platform validation study,” Int. J. Comput. Assist. Radiol. Surg. 17(5), 911–920 (2022). 10.1007/s11548-022-02583-6 35334043

